# Motoneuron Differentiation of Induced Pluripotent Stem Cells from SOD1G93A Mice

**DOI:** 10.1371/journal.pone.0064720

**Published:** 2013-05-28

**Authors:** Xiao-Li Yao, Cheng-Hui Ye, Qiang Liu, Jian-bo Wan, Jun Zhen, Andy Peng Xiang, Wei-Qiang Li, Yitao Wang, Huangxing Su, Xi-Lin Lu

**Affiliations:** 1 Department of Neurology, The First Affiliated Hospital, Sun Yat-sen University, Guangzhou, Guangdong, People's Republic of China; 2 Department of Geriatrics, The First Affiliated Hospital, Sun Yat-sen University, Guangzhou, Guangdong, People's Republic of China; 3 State Key Laboratory of Quality Research in Chinese Medicine, Institute of Chinese Medical Sciences, University of Macau, Macao, People's Republic of China; 4 Department of Rehabilitation Medicine, The Fifth Affiliated Hospital, Sun Yat-sen University, Zhuhai, Guangdong, People's Republic of China; 5 Center for Stem Cell Biology and Tissue Engineering, Key Laboratory for Stem Cells and Tissue Engineering, Ministry of Education, Sun Yat-Sen University, Guangzhou, Guangdong, People's Republic of China; Muséum National d'Histoire Naturelle, France

## Abstract

Amyotrophic lateral sclerosis (ALS) is a neurodegenerative disorder mainly affecting motor neurons. Mutations in superoxide dismutase-1 (SOD-1) account for about 20% of familial ALS patients. A robust supply of motoneurons carrying the mutated gene would help understand the causes of motoneuron death and develop new therapeutics for the disease. Here, we established induced pluripotent stem (iPS) cell lines from SOD1G93A mice and compared their potency in motoneuron generation with normal iPS cells and mouse embryonic stem cells (E14). Our results showed that iPS cells derived from SOD1G93A mice possessed the similar potency in neuronal differentiation to normal iPS cells and E14 cells and can be efficiently driven to motoneuron-like phenotype. These cells exhibited typical neuronal morphology, expressed key motoneuron markers, including ChAT and HB9, and generated repetitive trains of action potentials. Furthermore, these neurons highly expressed human SOD-1 and exhibited shorter neurites compared to controls. The present study provides evidence that ALS-iPS cells can be used as disease models in high-throughput screening and mechanistic studies due to their ability to efficiently differentiate into specific neuronal subtypes.

## Introduction

Amyotrophic lateral sclerosis (ALS) is an adult-onset neurodegenerative disease characterized by the selective loss of motoneurons in the cerebral cortex, brainstem, and spinal cord, leading to atrophy of limb, axial, and respiratory muscles [1]. Mutations in superoxide dismutase-1 (SOD-1) account for about 20% of familial ALS patients [2], [3]. SOD1G93A mice is a widely accepted model for the ALS research, which express mutant G93A of human SOD-1 and develop clinical symptoms similar to those seen in ALS patients [4]. Motoneurons from SOD1G93A mice could give some information to study the mechanism of ALS [5], [6]. A robust supply of motoneurons carrying the genes responsible for this condition would help understand the causes of motoneuron death in ALS and develop new therapeutics for the disease.

Recently, somatic cells can be reprogrammed to a pluripotent state through viral transduction of four transcription factors Oct4, Sox2, c-Myc, and Klf4 [7]–[9]. The induced pluripotent stem (iPS) cells were indistinguishable from ES cells in proliferative and developmental potential, and they can differentiate into derivatives of all germ layers. Several protocols have been developed to induce iPS cells to efficiently differentiate into neurons [10]–[14]. However, it remains unknown whether iPS cells with genetic deficiency possess neuronal differentiation potential similar to normal cells lines.

In this study, we compared the neuronal differentiation potential between iPS cells derived from SOD1G93A mice and iPS cells derived from normal C57BL/6 mice and investigated whether SOD1 mutations could influence the neuronal differentiation, especially motoneuron generation from iPS cells. Results of the present study would provide evidence on the possibility of the efficient generation of motoneurons from iPS cells with SOD mutations.

## Results

### Generation and characterization of iPS cells from tail-tip fibroblasts

**Figure 1:**
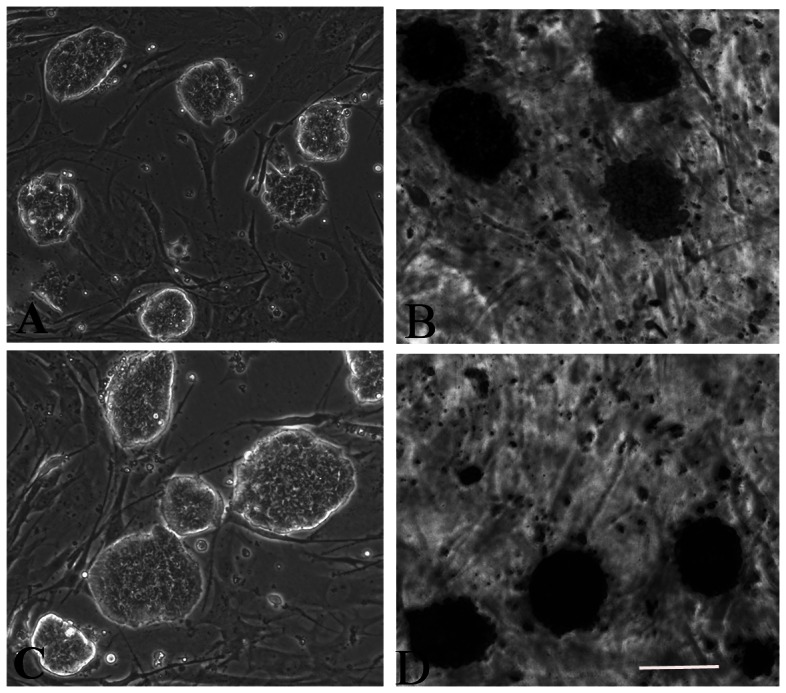
Establishment of mouse iPS cell lines from SOD1G93A mice and C57BL/6 mice. (A) Phase contrast image shows that iPS cells from SOD1G93A mice (ALS-iPS-1) grew as colonies on mitomycin-treated MEF feeder cells. (B) These clones exhibited high AP activity. (C) Phase contrast image shows that iPS cells from C57BL/6 mice grew as colonies on mitomycin-treated MEF feeder cells. (D) These clones exhibited high AP activity. Scale bar: 500 µm.

**Figure 2:**
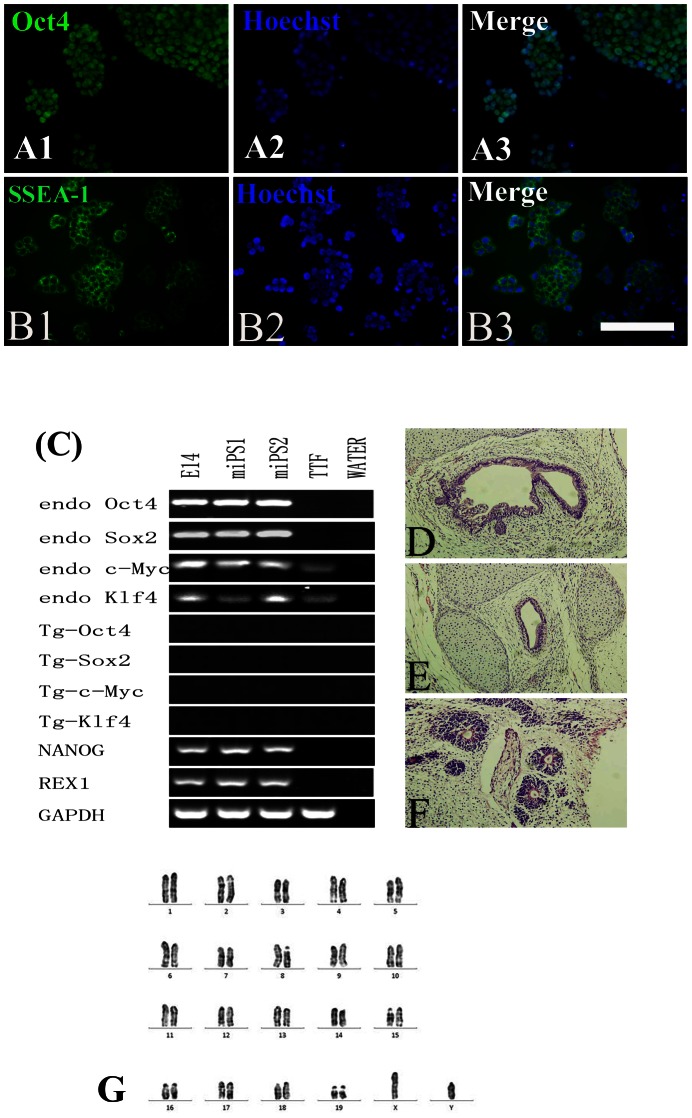
Immunostaining shows that the established iPS cell line (ALS-iPS-1) was positive for Oct4 (A) and SSEA-1 (B). (C) The expression patterns of pluripotent genes in iPS clones, E14 cells, and fibroblasts. The results revealed that all analyzed iPS clones induced expression from the endogenous Oct4, Sox2, and Nanog loci, and none of these genes were expressed in the original TTF fibroblasts. (D–F) Teratoma derived from ALS-iPS-1 cells contained cells belonging to all three germ layers, including endoderm-derived glandular (D), mesoderm-derived cartilage tissue (E), and ectoderm-derived neural tubes (F). Karyotype analyses demonstrated that ALS-iPS-1 clones showed a normal karyotype (G). Scale bar: 100 µm in A and B; 250 µm in D, E, and F.

Totally 6 iPS cell lines were generated by retroviral expression of mouse Oct4, Sox2, c-Myc, and Klf4 from B6SJL-TgN TTFs and C57BL/6 TTFs for characterization and comparison, in which 3 iPS cell lines were derived from 3 transgenic B6SJL-TgN mice (ALS-iPS) and 3 iPS cell line were derived from 3 C57BL/6 mice (C57-iPS) (Figs. 1A and 1C). To confirm that these iPS cells exhibit ES-like properties, we examined some ES cell markers that included alkaline phosphatase (AP) activity and ES cell-specific transcription factors Oct4 and SSEA-1. Results shown in Figs. 1B and 1D demonstrated that the iPS clones exhibited high AP activity. The selected iPS clones were also shown to be positive for Oct4 and SSEA-1 (Figs. 2A and 2B). To assess the gene expression pattern of the iPS clones, we isolated RNA from iPS cells and the result indicated that the endogenous Oct4, Sox2, c-Myc, Klf4, and Nanog were expressed which confirmed activation of these loci. Results shown in Fig. 2C demonstrated that the transgenes of selected clones from both ALS-iPS-1 and C57-iPS-12 cells were silenced. Importantly, all analyzed iPS clones induced expression from the endogenous Oct4, Sox2, and Nanog loci, and none of these genes were expressed in the original TTF fibroblasts, further supporting of successful reprogramming. Karyotype analyses demonstrated that all analyzed ALS-iPS-1 clones (Fig. 2G) and C57-iPS-12 clones (data not shown) exhibited a normal karyotype.

To confirm the pluripotency of the iPS cells, we injected iPS cells intramuscularly into nude mice. Teratomas formed 4–6 weeks after injection. HE staining of tumor sections from teratomas dissected 5 weeks after injection demonstrated the presence of cell types of all the 3 germ layers (Figs. 2D–F), including gland tissues (endoderm, Fig. 2D), cartilage tissue (mesoderm, Fig. 2E), and neural tube (ectoderm, Fig. 2F).

### Transduction and expression of Tα1 α-tubulin/hrGFP (Tα1) during iPS cell neuronal differentiation

To facilitate to monitor the neuronal differentiation potential, we transduced iPS cell lines (ALS-iPS-1 and C57-iPS-12) with lentivector containing Tα1 α-tubulin promoter. E14 cells were used as control. After antibiotic selection, tansduced undifferentiated mouse iPS cells and E14 cells were obtained.

**Figure 3:**
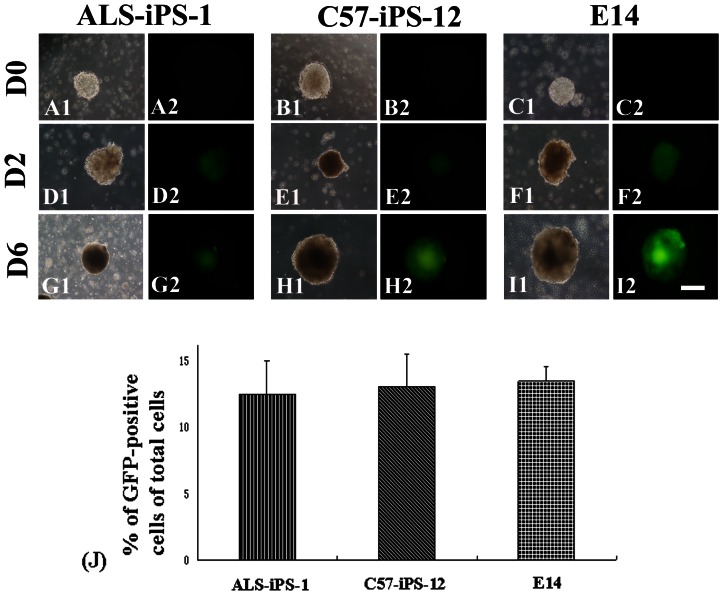
Live monitoring of Tα1 α-tubulin promoter driven GFP in the neural differentiation process. At day 0, undifferentiated ALS-iPS-1 (A), C57-iPS-12 (B) and E14 (C) did not express GFP. GFP expression appeared at day 2 of the EBs of ALS-iPS-1 (D), C57-iPS-12 (E) and E14 (F). The expression of GFP increased over time (G–I). (J) FACS analysis indicated that 12.4±2.6% of ALS-iPS-1 differentiated cells was GFP positive, 13.2±2.4% of C57-iPS-12 differentiated cells was GFP positive, while 13.1±1.8% of E14 differentiated cells was GFP positive cells, showing no statistical difference in the number of GFP-positive among the three cell lines (P>0.05). Scale bar: 75 µm.

**Figure 4:**
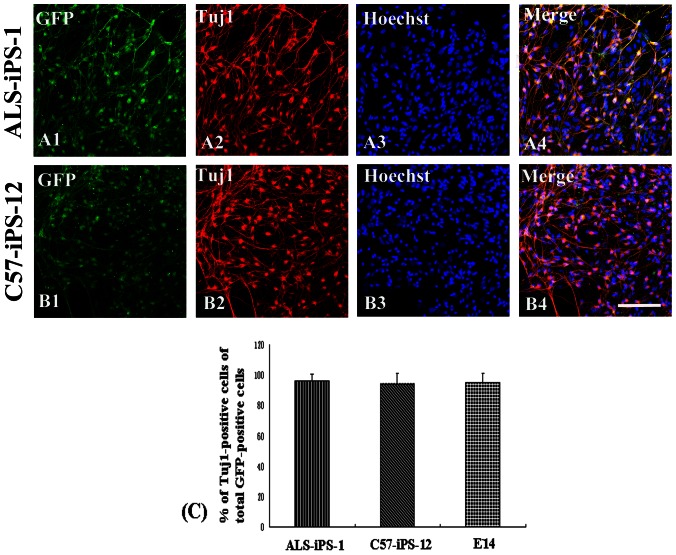
Immunocytostaining of differentiated neurons. FACS sorted GFP-positive cells were cultured on the poly-L-ornithin/laminin-coated plates for 24 hr, and then analyzed by immunofluorescence. The GFP-expressing cells were well overlapped with β-III-tubulin (Tuj1) immunolabeling. (A) ALS-iPS-1 cells. (B) C57-iPS-12 cells. (C). Quantification data demonstrated that 95.2±4.7% of the sorted cells from ALS-iPS-1 cells were stained positively for β-III-tubulin,94.4±6.5% of the sorted cells from C57-iPS-12 cells were stained positively for β-III-tubulin,while 95.0±5.6% from E14 cells were positive for β-III-tubulin, suggesting that nearly all GFP-positive cells were differentiated neurons. Scale bar: 80 µm.

Under the control of the Tα1 α-tubulin promoter, GFP expression appeared at day 2 of the EBs (Fig. 3). After 4 days treatment with RA, the expression of GFP increased over time (Fig. 3). To identify and separate neurons from differentiated E14 and iPS cells, we used a strategy of GFP-based FACS. FACS analysis indicated that 12.4±2.6% of ALS-iPS-1 differentiated cells was GFP positive, 13.2±2.4% of C57-iPS-12 differentiated cells was GFP positive, while 13.1±1.8% of E14 differentiated cells was GFP positive cells, showing no statistical difference in the number of GFP-positive among the three cell lines (P>0.05) (Fig. 3J). After fluorescent-activated cell sorting, the GFP positive cells were enriched to high purity. The sorted GFP-expressing cells were stained positively for β III-tubulin. Quantitative analysis of these immunocytostaining experiments demonstrated that 95.2±4.7% (403/423, n = 6) of the sorted cells from ALS-iPS-1 cells were stained positively for β-III-tubulin, 94.4±6.5% (425/450, n = 6) of the sorted cells from C57-iPS-12 cells were stained positively for β-III-tubulin,while 95.0±5.6% (419/441, n = 6) from E14 cells were stained positively for β-III-tubulin, suggesting that nearly all GFP-positive cells were differentiated neurons (Fig. 4).

### Motoneuron differentiation and characterization of iPS cell lines

**Figure 5:**
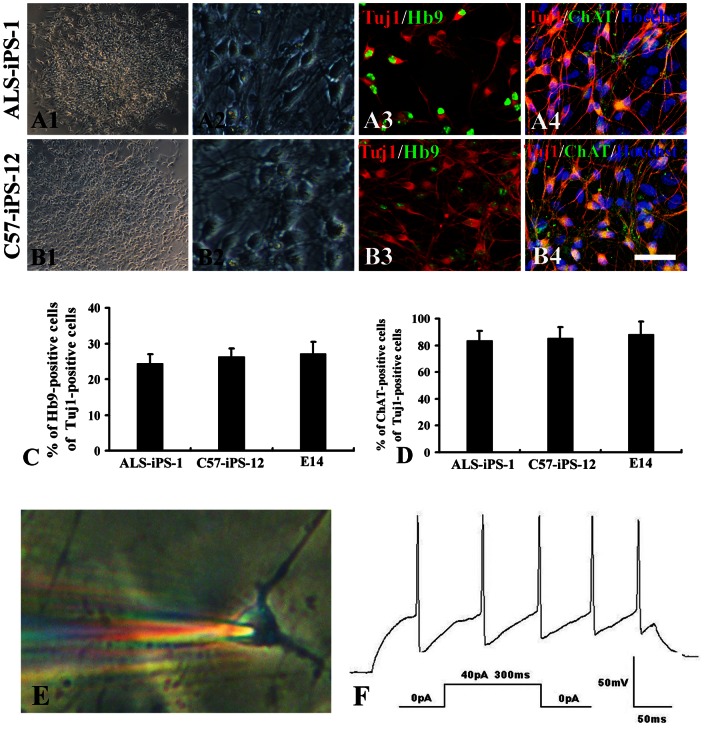
Motoneuron differentiation and characterization. (A). Motoneuron differentiation of ALS-iPS-1. (B). Motoneuron differentiation of C57-iPS-12. (A1 and B1) Phase contrast image shows neuronal differentiation of after 5 days treatment with RA and SHH. (A2 and B2) Higher magnification shows typical neuronal morphology. (A3 and B3) Confocal imaging shows that HB9 was expressed on differentiation to TuJ1-positive neurons. (A4 and B4) Confocal imaging shows that the majority of mouse iPSC-derived Tuj-1-positive neurons co-expressed ChAT. (C). Quantification data show that there is no difference in the percentage of Hb9-positive neurons across the iPS cell lines and ES cell line (P>0.05). (D) Quantification data show that there is no difference in the percentage of ChAT-positive neurons across the iPS cell lines and ES cell line (P>0.05). (E). A phase contrast image showing that a whole-cell patch-clamp recording electrode attached on an iPSC-derived motoneuron-like cell under an IR/DIC microscopy. (F). Single current injection (300 ms duration, 40 pA) showing these motoneurons generated repetitive trains of action potentials. Scale bar: 200 µm in A1 and B1; 80 µm in A2–A4; B2–B3.

**Figure 6:**
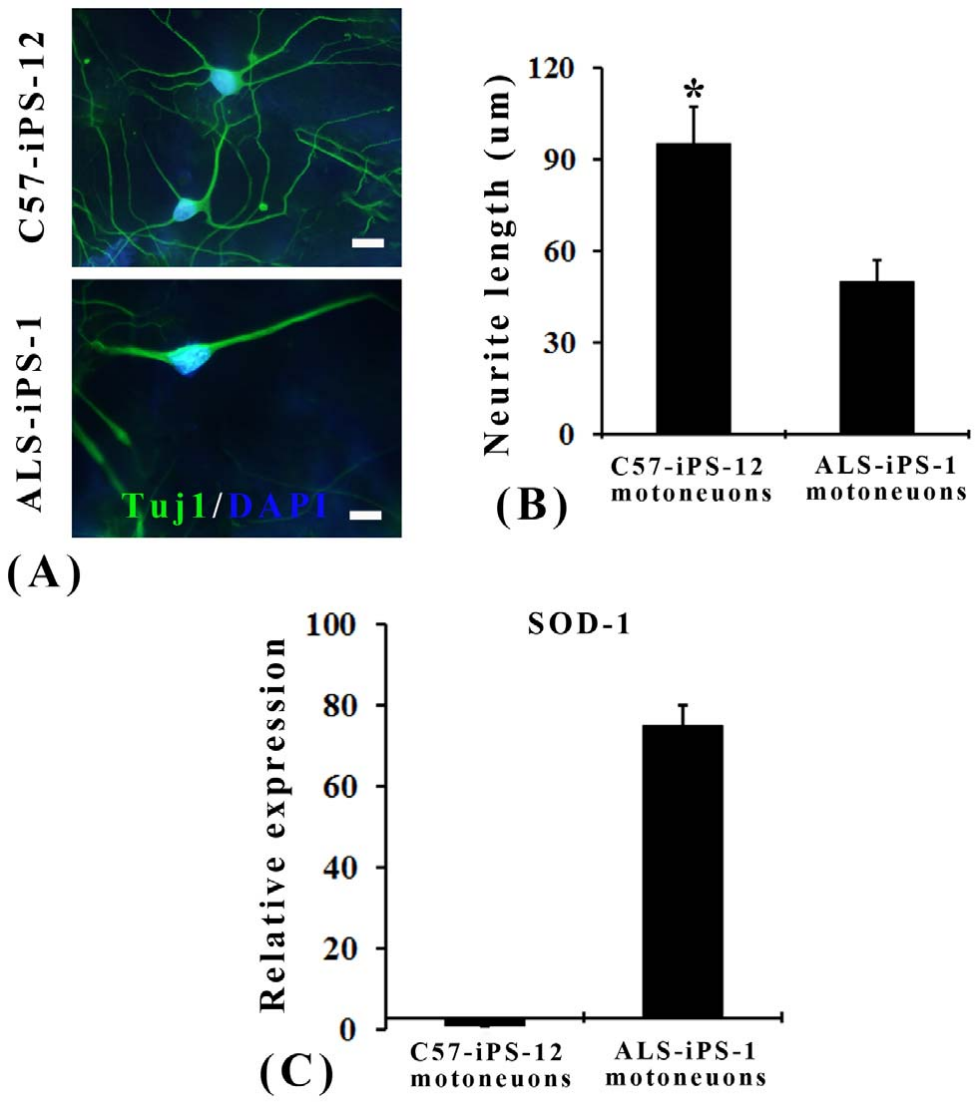
Phenotypes of ALS-iPS-derived motoneurons. (A). Micrographs showing that ALS-iPS cells-derived motoneurons exhibited shorter neuritis than C57-iPS cells-derived motoneurons. (B). Quantification data show that the length of neurites of ALS-iPS-derived motoneurons is 50.5±7.9 µm, which is significantly shorter than 95.8±12.5 µm for C57-iPS cells-derived motoneurons (P<0.001, student t-test). (C). Quantitative real time RT-PCR showed that these ALS-iPS cells-derived motoneurons highly expressed human SOD-1, while C57-iPS cells-derived did not express any. Scale bar: 15 µm.

Incubation with RA and SHH led to efficient motoneuron generation from iPS and E14 cell lines. Approximately 83.5±7.9% β-III-tubulin-positive neurons co-expressed ChAT in ALS-iPS-1 cell culture after 5 days treatment with RA and SHH (Fig. 5A4). When stained with Hb9 (a specific postmitotic motoneuron marker), 24.3±2.7% Tuj1-positive neurons were found to be Hb9-positive (Fig. 5A3). C57-iPS-12 cells showed similar potency in motoneruon generation. 85.2±6.8% β-III-tubulin-positive neurons co-expressed ChAT (Fig. 5B4) and 26.1±3.4% β-III-tubulin-positive neurons co-expressed Hb9 (Fig. 5B3) in C57-iPS-12 cell culture after 5 days treatment with RA and SHH. There is no difference in the percentage of both ChAT-positive neurons and Hb9-positive neurons between ALS-iPS-1 and C57-iPS-12 groups (ChAT: 83.5±7.9% vs 85.2±6.8%, P>0.05; Hb9: 24.3±2.7% vs 26.1±3.4%, P>0.05), suggesting that SOD1 mutation did not reduce the potency of motoneuron differentiation compared to the normal iPS cell line. Efficient motoneuron generation from E14 cells was also observed. Five days treatment with RA and SHH led to 88.6±9.4% β-III-tubulin-positive neurons co-expressing ChAT and 27.2±3.1% β-III-tubulin-positive neurons co-expressing Hb9 (Figs. 5C and D). The three cell lines did no differ in the potency of motoneuron generation as revealed by the percentage of ChAT-positive neurons and Hb9-positive neurons (Figs. 5C and D). Standard whole-cell patch clamp, current-clamp techniques were then used to study the electrical properties of these motor neurons (Fig. 5E). Both ALS-iPS and C57-iPS cells-derived motoneurons generated repetitive trains of action potentials, suggesting that they were functional (Fig. 5F). However, ALS-iPS cells-derived motoneurons exhibited shorter neuritis (50.5±7.9 µm compared to 95.8±12.5 µm for C57-iPS cells-derived motoneurons, P<0.001, student t-test) (Figs. 6A and B). Quantitative real time RT-PCR showed that these ALS-iPS cells-derived motoneurons highly expressed human SOD-1, while C57-iPS cells-derived did not express any (Fig. 6C).

## Discussion

Using iPSCs technology, researchers can achieve ES-like cells without the ethical dilemma. The derivation of iPS cells is of such great importance because of the ease and reproducibility of generating them. Direct reprogramming provides, for the first time, a realistic way of generating sufficient numbers of patient-specific pluripotent stem cells. Such cells could be used for regenerative and therapeutic purposes, as demonstrated in mouse models of, for example, sickle cell anemia and Parkinson's disease, respectively [15], [16]. In this study, we generated iPS cells from TTFs of SOD1G93A mice by retroviral constructs encoding Oct4, Sox2, c-Myc and Klf4 and demonstrated that they possessed the similar potency in neuronal differentiation to normal iPS cells and mouse ESCs (E14), providing evidence that they can be used as disease models in high-throughput screening and mechanistic studies.

We used a neuron-specific promoter to monitor the neural differentiation processes of iPS cells. After antibiotic selection, mouse iPS cells and ESCs expressing the Tα1: hGFP with a high degree of purity and stability could be obtained. Our data have shown that it can be used in studies to monitor the differentiation of pluripotent stem cells to early neurons. Modification of derivatives with fluorescent markers allows for the cell separation under a fluorescence-equipped dissecting microscope or by fluorescence activated cell sorting. More than 95% GFP-positive cells were found to be Tuj1-positive neurons across iPS cell lines and one ES cell line, confirming the validity of the T**α**1 α–tubulin promoter-based neuronal selection strategy.

The ability to generate iPS cells and efficiently differentiate them into specific neuronal subtypes will provide powerful new tools to study complex neurogenetic disorders. ALS-iPS cell-derived motoneurons are an ideal model to study the disease, because they contain the same nuclear genome as of patients, which helps understand the cellular physiology of the disease and development of drug treatment. Using an established protocol, ALS-iPS cells could be driven to motoneuron-like phenotypes efficiently. Our results were supported by the previous findings using SOD1G93A mES cells that the presence of G93A hSOD1 mutation does not affect early neuron differentiation [17]. These cells expressed key motoneuron markers, including ChAT and HB9, indicating the usefulness of these cells in drug screens and basic research. A key limitation of the study is that no phenotypic changes that may correspond to the disease process were described and future work is also needed to functionally characterize these derived cells for basic neuronal properties.

## Conclusion

Here, we reported a successful derivation of mouse iPS cells from SOD1G93A mice and these iPS cells can be induced to differentiation to motorneurons with efficiency similar to that of mouse ES cells and wild type mouse iPS cells. Our preliminary study provides a new ALS disease models for high-throughput screening and mechanistic studies.

## Materials and Methods

### Cells culture

All experimental procedures were approved by the Institutional Animal Ethical Committee of Sun Yat-sen University and were conducted according to the Guide for the Care and Use of Laboratory Animal of the National Institute of Health (Publication No. 80–23, revised 1996).

Fibroblasts were prepared from tail tips of transgenic male mice B6SJL-TgN (SOD1G93A) 1 Gur (G93A; Jackson Laboratories) and adult male C57BL/6 mice as reported [7]. Tail tip fibroblasts (TTFs) were maintained in DMEM containing 10%FBS and expanded in 1∶5 ratio. Murine cell line E14 is purchased from ATCC, American Type Culture Collection (Rockville, MD, USA). iPS cells were cultured in DMEM medium supplemented with 15% FBS, 2 mM glutamine, 1% non-essential amino acids, 50 units/50 mg/ml penicillin-streptomycin and 0.1 mM b-mercaptoethanol (all Invitrogen), and 10 ng/ml LIF (Chemicon, Temecula, California, USA) on gamma irradiation-treated mouse embryonic fibroblast (MEF) feeders at 37°C with 5% CO2 in air.

### Retroviral infection and iPS cell induction

The protocol for mouse iPS was approved by the Institutional Animal Ethical Committee of Sun Yat-sen University.

Retroviral infection was performed as described by others [7], [8] with minor modifications. Plat-E cells are purchased from Cell Biolabs (San Diego, CA 92126 USA). Plat-E cells were seeded at 1×10^6^ cells per well of a 6-well plate. Next day, the cells were transfected with pMXs vectors carrying Oct4, Sox2, c-Myc and Klf4 cDNAs using Lipofectamine 2000 (Invitrogen) according to the manufacturer's instructions. After 12 hr, the medium was replaced with fresh medium. Virus-containing supernatants were collected from each plate at 72 hr post-transfection, filtered through a 0.45 µm filter before transduction. Equal volumes of the supernatants were mixed and supplemented with 4 µg/ml polybrene. TTFs were seeded at a density of 5×10^4^ cells per 6-well plate and incubated in the virus/polybrene-containing supernatants for 24 hr. Four days after infection, the cells were further subcultured on irradiated MEFs in ES medium containing LIF. About two weeks after infection, the colonies were mechanically isolated, and propagated under ES conditions.

### Cell line characterization

Alkaline phosphatase staining, immunofluorescence microscopy, semi-quantitative RT-PCR for transgene integration, karyotyping, and teratoma formation were carried out to characterize iPS cell lines. Direct Alkaline phosphatase (AP) activity was analyzed with the alkaline phosphatase substrate BCIP/NBT (Sigma) according to the manufacturer's guidelines. The following primary antibodies were used: anti-Oct-4 (Santa Cruz Biotechnology, Santa Cruz, CA, USA), SSEA1 (Chemicon). Total mRNA was isolated using TRIZOL and 1 µg was used to synthesize cDNA using Murine Leukemia Virus reverse transcriptase (Fermentas) and oligo-dT primers (Fermentas) according to the manufacturer's instruction. Then the samples were subjected to amplification with mouse specific primers. β-actin was used as positive control. The PCR products were analyzed by 1.2% agarose gel electrophoresis and visualized by ethidium bromide staining. The detailed information of primers is listed in Table 1. To form teratomas, approximately 2×10^6^ cells were injected into hind limb muscle of 5-week-old nude mice. After five weeks, teratomas were dissected and fixed in 4% paraformaldehyde. Samples were embedded in paraffin and processed with hematoxylin and eosin staining.

**Table 1:** 

Gene	Sequence (5′ to 3′)
Endo-Oct4	CTTCTGCTTCAGGAGCTTGG
	GAAGGAGAAGCTGGAGCAAA
pMX-Oct4	CCCCAGGGCCCCATTTTGGTACC
	TTATCGTCGACCACTGTGCTGCTG
Endo-Sox2	GGGAAATGGGAGGGGTGCAAAAGG
	TTGCGTGAGTGTGGATGGGATTGGG
pMX-Sox2	GGCACCCCTGGCATGGCTCTTGGCTC
	TTATCGTCGACCACTGTGCTGCTG
Endo-c-Myc	GCGTCCTGGGAAGGGAGATCCGGAC
	TTGAGGGGCATCGTCGCGGGAGGCG
pMX-c-Myc	CAACAACCGAAAATGCACCAGCCCC
	TTGCGTGAGTGTGGATGGGATTGGG
Endo-Klf4	TGATTGTAGTGCTTTCTGGCTGGGCC
	ACGATCGTGGCCCCGGAAAAGGACC
pMX-Klf4	ACGATCGTGGCCCCGGAAAAGGACC
	TTGAGGGGCATCGTCGCGGGAGGCG
Nanog	CCTATGCCTGTGATTTGTGGG
	AGGTTGTTTGCCTTTGGGAC
Rex1	CAGATCCTAAACAGCTCGCAGAAT
	GCGTACGCAAATTAAAGTCCAGA
GAPDH	GATTTGTGGGCCTGAAGAAA
	TGTAGACCATGTAGTTGAGGTCA

### Vector construction and lentivirus production

To generate Lentivirus, 293FT cells (Invitrogen) were transfected at 90% confluence using Lipofectamine 2000 (Invitrogen). For a 10 cm plate, 9 µg of ViraPower packaging mix and 3 µg of pLV/Final-puro-Tα1 α-tubulin-hrGFP [18] lentiviral vector were co-transfected into the 293FT cells using 36 µl Lipofectamine 2000 reagent. Virus supernatant was collected 72 hours later and filtrated through 0.45-µm filters.

### ES and iPS cell transduction and drug selection

E14 (American Type Culture Collection, ATCC) and iPS cells were transduced for 12 h with lentivirus in the presence of 6 µg/ml of polybrene. Puromycin selection (1–3 µg/mL) was started 96 hr post-transduction and lasted for seven days.

### Neural differentiation

Undifferentiated E14 cells and iPS cells were treated with 0.125% trypsin-EDTA and grown in aggregate cultures for 2 days in DFK10 medium to form embryoid bodies (EBs). DFK10 medium consisted of DMEM/F12 (GIBCO, Invitrogen), knock-out serum replacement (10%), penicillin/streptomycin (1%), N2 supplement (2.4%), glucose (4.5 mg/ml), L-glutamine (200 mM), heparin (1 u/µl; Sigma), and β-mercaptoethanol (0.1 mM). After 2 days, EBs were treated with 1 µM of all-trans RA (Sigma) for another 4 days. These EBs with 4 days RA treatment were subsequently used for FACS sorting studies.

For motoneuron differentiation, SHH and RA were used as described previously with slight modifications [19], [20]. After 2 days culture of dissociated E14 or iPS cells in DFK10 medium to form embryoid bodies (EBs), RA (0.1 µM; Sigma) and SHH (200 ng/ml; R&D Systems) were added to culture for additional 5 days. To facilitate immunostaining analysis, EBs were plated on laminin-coated coverslips in DFK10 medium at day 5 and subsequently supplemented with 10 ng/mL BDNF, 10 ng/mL GDNF, 10 ng/mL CNTF, and 10 ng/mL IGF (R&D Systems) to aid in neuronal survival.

### Motoneuron characterization

Immunocytochemical staining on coverslip cultures was performed to characterize neuronal differentiation. Motoneurons were double-stained with mouse anti-Tuj1 (1∶500, Sigma) and goat anti-ChAT (1∶400, Millipore) or rabbit anti-Tuj1 (1∶200, Sigma) and mouse anti-HB9 (1∶50, DSHB). Species-specific fluorescence-conjugated secondary antibodies conjugated to Alexa 568 or 488 (1∶400, Molecular Probes) were applied for 2 h at room temperature. Cell slides were then counterstained with DAPI to stain nuclei, and cover-slipped with antifade mounting media (FluorSave, Calbiochem). The images were taken with a laser confocal microscope (LSM510 META, Carl Zeiss Meditec).

Whole-cell patch-clamp recording was used to study the electrophysiological properties of both ALS-iPS and C57-iPS derived motoneurons in culture. Patch pipettes (resistance 3–5 MΩ) were filled with the following (in mM): 140 potassium methanesulfonate, 10 HEPES, 5 NaCl, 1 CaCl2, 0.2 EGTA, 3 ATP-Na2, 0.4 GTP-Na2, pH 7.3 (adjusted with KOH). The external solution contained (in mM): 120 NaCl, 1.2 KH2PO4, 1.9 KCl, 26 NaHCO3, 2.2 CaCl2, 1.4 MgSO4, 10 D-glucose, 7.5 HEPES (pH with NaOH to 7.3). The bath solution was equilibrated with 95% O2 and 5% CO2 before use. Resting potentials were maintained at about −65 mV. Whole-cell patch-clamp recordings were amplified and filtered using an Axopatch 200B amplifier (Molecular Devices, Sunnyvale, CA). Signals were sampled at 10 kHz using a Digidata 1440A analog-to-digital converter and acquired and stored on a computer hard drive using pClamp10 software. All voltage and current-clamp recordings were performed. Data were analyzed using pClamp10 (Clampfit).

Total RNAs of motoneurons derived from ALS-iPS and C57-iPS cells were extracted using TRIzol (Invitrogen) respectively. Quantitative real time RT-PCR (qPCR) was performed using a Thermal Cycler DiceTM Real Time System and SYBR Premix EX TaqTM (Takara). The primer for human SOD1 (Forward 5′- CAT CAG CCC TAA TCC ATC TGA-3′ and Reverse 5′- CGC GAC TAA CAA TCA AAG TGA-3′) was used. β-actin was used for qPCR normalization, and all items were measured in triplicate.

### FACS sorting

For flow cytometry analysis, differentiated cells on day 6 were trypsinized and filtered through a 40 µm nylon mesh to remove cell debris. The cells were resuspended in PBS at a concentration of 1×10^6^ cells/ml. Cell analysis and sorting were performed on a FACS-Aria (BD-Biosciences), through which cell sorting purity of >97% was achieved consistently. An aliquot of untransfected E14 cell suspension was used as negative control. The cells were analyzed by light forward and side scatter by a 488-nm laser beam. Sorting procedures were only based on fluorescence intensity and performed with a flow rate of 1500 events/sec. GFP-positive cells were replated on poly-L-ornithin/laminin-coated dishes in N2B27 medium for 24 hr and were then analyzed for β-III-tubulin expression.

### Statistical analysis

Five wells per experiment were imaged for quantification. Results are the average ± SEM of data from a minimum of three experiments unless stated otherwise. Around 200 cells were counted for each marker. Statistical analysis was performed using one way ANOVA. In case of small numbers in the contingency table, a two-tailed Fisher's-exact test was used.
