# Correction: Motoneuron Differentiation of Induced Pluripotent Stem Cells from SOD1G93A Mice

**DOI:** 10.1371/annotation/2033887d-1abc-48d1-b87f-dc30f75cfa2d

**Published:** 2013-07-01

**Authors:** Xiao-Li Yao, Cheng-Hui Ye, Qiang Liu, Jian-bo Wan, Jun Zhen, Andy Peng Xiang, Wei-Qiang Li, Yitao Wang, Huangxing Su, Xi-Lin Lu

The name of the ninth author was spelled incorrectly. The correct name is: Huangxing Su.

